# Supplementary material for: Presence of Trypanosoma cruzi (TcI) in different tissues of Didelphis virginiana from the metropolitan area of Merida, southeastern Mexico: Epidemiological relevance and implications for non-vector transmission routes
Source: PLoS Negl Trop Dis. 2024 Dec 13;18(12):e0012733. doi: 10.1371/journal.pntd.0012733 (PMC11676564; doi:10.1371/journal.pntd.0012733)
Supplement: S1 File — (DOCX) [file pntd.0012733.s002.docx]

**S1 File.** Mitochondrial Cytochrome b (Cytb) haplotype sequences of *Didelphis virginiana*.

> CytbHap1

TTGATTTACCAACACCATCTAACATCTCAGCTTGATGGAATTTTGGTTCACTATTAGGAGTGTGCCTAATTATTCAAATCCTTACAGGCTTATTCTTAGCAATACATTACACATCTGACACATCAACCGCATTTTCATCAGTAGCCCATATTTGCCGAGACGTAAACTACGGATGACTTATCCGAAATATCCACGCCAACGGAGCATCTATATTCTTTATATGCCTCTTCCTTCATGTAGGACGAGGAATCTATTACGGATCATACCTTTACAAAGAAACATGAAATATTGGAGTTATCCTACTATTAACAGTTATAGCTACTGCATTCGTTGGCTACGTTCTACCATGAGGACAAATATCATTTTGAGGCGCAACGGTTATTACTAACTTATTATCTGCCATCCCATATATCGGAAGTACACTAGTAGAATGAATTTGAGGAGGATTCTCCGTTGATAAAGCTACACTAACTCGATTTTTTGCTTTTCACTTTATTCTTCCATTCATCATTTTAGCTATAGTAGTAGTAC

> CytbHap2

TTGATCTACCAACACCATCTAACATCTCAGCTTGATGGAATTTTGGTTCACTATTAGGAGTGTGCCTAATTATTCAAATTCTTACAGGCTTATTCTTAGCAATACATTACACATCTGACACATCAACCGCATTTTCATCAGTAGCCCATATTTGCCGAGACGTAAACTACGGATGACTTATCCGAAATATCCACGCCAACGGAGCATCTATATTCTTTATATGCCTCTTCCTTCATGTAGGACGAGGAATCTATTACGGATCATACCTTTACAAAGAAACATGAAATATTGGAGTTATCCTACTATTAACAGTTATAGCTACTGCATTCGTTGGCTACGTTCTACCATGAGGACAAATATCATTTTGAGGCGCAACGGTTATTACTAACTTATTATCTGCCATCCCATATATCGGAAGTACACTAGTAGAATGAATTTGAGGAGGATTCTCCGTTGATAAAGCTACACTAACTCGATTTTTTGCTTTTCACTTTATTCTTCCATTCATCATTTTAGCTATAGTAGTAGTAC

> CytbHap3

TTGATCTACCAACACCATCTAACATCTCAGCTTGATGGAATTTTGGTTCACTATTAGGAGTGTGCCTAATTATTCAAATCCTTACAGGCTTATTCTTAGCAATACATTACACATCTGACACATCAACCGCATTTTCATCAGTAGCCCATATTTGCCGAGACGTAAACTACGGATGACTTATCCGAAACATCCACGCCAACGGAGCATCTATATTCTTTATATGCCTCTTCCTTCATGTAGGACGAGGAATCTATTACGGATCATACCTTTACAAAGAAACATGAAATATTGGAGTTATCCTACTATTAACAGTTATAGCTACTGCATTCGTTGGCTACGTTCTACCATGAGGACAAATATCATTTTGAGGCGCAACAGTTATTACTAACTTATTATCTGCCATCCCATATATCGGAAGTACACTAGTAGAATGAATTTGAGGAGGATTCTCCGTTGATAAAGCTACACTAACTCGATTTTTTGCTTTTCATTTTATTCTTCCATTCATCATTTTAGCTATAGTAGTAGTAC

> CytbHap4

TTGATCTACCAACACCATCTAACATCTCAGCTTGATGGAATTTTGGTTCACTATTAGGAGTGTGCCTAATTATTCAAATCCTTACAGGCTTATTCTTAGCAATACATTACACATCTGACACATCAACCGCATTTTCATCAGTAGCCCATATTTGCCGAGACGTAAACTACGGATGACTTATCCGAAATATCCACGCCAACGGAGCATCTATATTCTTTATATGCCTCTTCCTTCATGTAGGACGAGGAATCTATTACGGATCATACCTTTACAAAGAAACATGAAATATTGGAGTTATCCTACTATTAACAGTTATAGCTACTGCATTCGTTGGCTACGTTCTACCATGAGGACAAATATCATTTTGAGGCGCAACGGTTATTACTAACTTATTATCTGCCATCCCATATATCGGAAGTACACTAGTAGAATGAATTTGAGGAGGATTCTCCGTTGATAAAGCTACACTAACTCGATTTTTTGCTTTTCACTTTATTCTTCCATTCATCATTTTAGCTATAGTAGTAGTAC

> CytbHap5

TTGATCTACCAACACCATCTAACATCTCAGCTTGATGGAATTTTGGTTCACTATTAGGAGTGTGCCTAATTATTCAAATCCTTACAGGCTTATTCTTAGCAATACATTACACATCTGACACATCAACCGCATTTTCATCAGTAGCCCATATTTGCCGAGACGTAAACTACGGATGACTTATCCGAAATATCCACGCCAACGGAGCATCTATATTCTTTATATGCCTCTTCCTTCATGTAGGACGAGGAATCTATTACGGATCATACCTTTACAAAGAAACATGAAATATTGGAGTTATCCTACTATTAACAGTTATAGCTACTGCATTCGTTGGCTACGTTCTACCATGAGGACAAATATCATTTTGAGGCGCAACGGTTATTACTAACTTATTATCTGCCATCCCATATATCGGAAGTACACTAGTAGAATGAATTTGAGGAGGATTCTCCGTTGACAAAGCTACACTAACTCGATTTTTTGCTTTTCACTTTATTCTTCCATTCATCATTTTAGCTATAGTAGTAGTAC

> CytbHap6

TTGATCTACCAACACCATCTAACATCTCAGCTTGATGGAATTTTGGTTCACTATTAGGAGTGTGCCTAATTATTCAAATCCTTACAGGCTTATTCTTAGCAACACATTACACATCTGACACATCAACCGCATTTTCATCAGTAGCCCATATTTGCCGAGACGTAAACTACGGATGACTTATCCGAAATATCCACGCCAACGGAGCATCTATATTCTTTATATGCCTCTTCCTTCATGTAGGACGAGGAATCTATTACGGATCATACCTTTACAAAGAAACATGAAATATTGGAGTTATCCTACTATTAACAGTTATAGCTACTGCATTCGTTGGCTACGTTCTACCATGAGGACAAATATCATTTTGAGGCGCAACGGTTATTACTAACTTATTATCTGCCATCCCATATATCGGAAGTACACTAGTAGAATGAATTTGAGGAGGATTCTCCGTTGATAAAGCTACACTAACTCGATTTTTTGCTTTTCACTTTATTCTTCCATTCATCATTTTAGCTATAGTAGTAGTAC

> CytbHap7

TTGATCTACCAACACCATCTAACATCTCAGCTTGATGGAATTTTGGTTCACTATTAGGAGTGTGCCTAATTATTCAAATCCTTACAGGCTTATTCTTAGCAATACATTACACATCTGACACATCAACCGCATTTTCATCAGTAGCCCATATTTGCCGAGACGTAAACTACGGATGACTTATCCGAAATATCCACGCCAACGGAGCATCTATATTCTTTATATGCCTCTTTCTTCATGTAGGACGAGGAATCTATTACGGATCATACCTTTACAAAGAAACATGAAATATTGGAGTTATCCTACTATTAACAGTTATAGCTACTGCATTCGTTGGCTACGTTCTACCATGAGGACAAATATCATTTTGAGGCGCAACGGTTATTACTAACTTATTATCTGCCATCCCATATATCGGAAGTACACTAGTAGAATGAATTTGAGGAGGATTCTCCGTTGATAAAGCTACACTAACTCGATTTTTTGCTTTTCACTTTATTCTTCCATTCATCATTTTAGCTATAGTAGTAGTAC
