# Supplementary material for: Presence of Trypanosoma cruzi (TcI) in different tissues of Didelphis virginiana from the metropolitan area of Merida, southeastern Mexico: Epidemiological relevance and implications for non-vector transmission routes
Source: PLoS Negl Trop Dis. 2024 Dec 13;18(12):e0012733. doi: 10.1371/journal.pntd.0012733 (PMC11676564; doi:10.1371/journal.pntd.0012733)
Supplement: Attachment — Submitted filename: Response to Reviewers.docx [file pntd.0012733.s003.docx]

***Reviewer: #1***

***Comments to the Author***

**Methods**

**Methods of opossum collection need to be more detailed. Were animals placed under sedation with chemical restraint? What does manually mean?**

We now detail better the methods of opossum collection. No chemical restraint was used when opossums were manually collected. The following text was added (lines 146 to 155): “Animals were collected by homeowners in the vicinity of their homes in environments such as backyards, parks and waste dumps and transported to the FMVZ of the UADY in cardboard boxes or pet carriers designed for dogs or cats. Additionally, other opossums were captured in the vicinity of some homes following reports to the SDS. In these cases, some animals were picked up manually by the research team, handled by grasping the tail with one hand and securing the head dorsally at the neck with the other before being placed in transport boxes, while others were captured using Tomahawk traps (66 x 23 x 23 cm, Tomahawk Live Trap Co.) baited with seasonal fruits (pineapple, sapota, or banana)”.

**Which portions of Merida were the opossums collected? I also suggest a map of the region and collection points as it relates to the urbanized setting. Were they collected in the city central or on the outskirts of Merida? A GIS map showing collection sites and possibly opossum collection density is also important.**

The opossums were collected from urban settings within the metropolitan area of Mérida, including the municipalities of Merida (n = 98), Uman (n = 3), and Conkal (n = 1). Details of sampling locations are now provided in Supplementary Table S1 and in the main text in the Results Section (Lines 290–291). Additionally, as recommended, we now include a map (Fig 1) indicating the sampling sites within the metropolitan area of Merida. The map was generated using RStudio (version 2023.06.1), integrating geolocation data collected during the study along with publicly accessible municipal boundary data for Yucatan, Mexico, obtained from INEGI (https://www.inegi.org.mx/temas/mg/). This information was used in compliance with the “Terms of Free Use of INEGI Information” (https://www.inegi.org.mx/inegi/terminos.html).

**I would also include how many were collected by homeowners, "manually" and tomahawk trap.**

We now provide the information requested in the Supplementary Table S1 and in the Results Section (Lines 292-294).

**Were animals released after biospecimen collection?**

After sample collection, opossums were released in a semi-rural area in the southern outskirts of Merida, unless they presented a condition incompatible with relocation, such as *T. cruzi* infection or severe injuries jeopardizing their survival. This information has now been added in the Methods Section (Lines 196-199).

**Were all animals alive at time of biospecimen collection? I ask this question as some animals were captured by homeowners. Did they put them in a cage?**

All animals were alive at the time of biospecimen collection. Those transported by homeowners were carried in cardboard boxes or pet carriers (designed for dogs or cats). We added the latter information in the Methods Section (Lines 146-149). Additionally, we now detail better how the sample collection was done in the Methods Section (Lines 179-183). With that information, together with the one we added regarding specimen release after sample collections to answer the previous comment (Lines 196-199), we feel that it is not necessary to precise that animals were alive at the time of sample collection in the main text.

**Did authors measure the amount of anal gland secretion and milk collected? Was this consistent between animals? Low volumes could explain some discrepancies in detection.**

We measured the amount of anal gland secretions and milk collected. The volumes collected ranged from 0.05 mL to 1 mL. However, for DNA extraction, we used the same amount for each opossum specimen: 100 µL for milk samples and 25 mg for anal gland secretions. We added this information in the Methods Section (Lines 195-196 for amount sampled and 216-218 for amount used for DNA extraction).

**Results**

**In the results section I also recommend a table or figure showing *T. cruzi* infected opossum as it relates to the city of Merida. A figure is preferred. This gives the reader a visual aspect to the findings. T. cruzi is being transmitted within an urbanized setting in a city with close to 1 million people. This is an important finding. As mentioned by the authors, there is growing evidence of zoonotic transmission to other hosts and humans via the anal gland secretions. Albeit, in this sample the detection was low, it was detected and has been detected in other studies.**

We thank the reviewer for this comment. We have now included a map (Fig 1C) to give the reader a visual aspect of the findings. The map indicates the urban sampling sites in the metropolitan area of Merida, and if the opossums collected in each site were positive or negative for *T. cruzi*. The map was generated using RStudio (version 2023.06.1), integrating geolocation data collected during the study along with publicly accessible municipal boundary data for Yucatan, Mexico, obtained from INEGI (https://www.inegi.org.mx/temas/mg/). This information was used in compliance with the “Terms of Free Use of INEGI Information” (https://www.inegi.org.mx/inegi/terminos.html).

**Conclusions**

**In the Discussion section I would also suggest adding:**

**I would suggest some discussion for blood meal analysis of *T. dimidiata* that have been collected in Merida and close to or near the study sites. It appears that most studies have shown bugs collected outside the city or close to the city? This author team has done a lot of this work but it would important to illustrate whether infected triatomines are found in the landscape of these collected opossums. There important potential discussion point here which could suggest that *T. cruzi* is being transmitted from opossum to opossum without the need for the vector.**

We thank Reviewer#1 for this suggestion. We have now incorporated findings from previous research on triatomine feeding sources in Yucatan (lines 382–384), as well as data on triatomine occurrence and prevalence of infection with *T. cruzi* in Merida (lines 377–379), and we enhanced the Discussion section by including better our results in the context of these other studies (Lines 382-387).

**I would mention this study in the Discussion:**

<https://pubmed.ncbi.nlm.nih.gov/37956445/>

We thank the reviewer for the suggestion. We have now incorporated and cited the article in the discussion section, as it reinforces the importance of opossums in non-vectorial transmission mechanisms of *T. cruzi* (Line 365, Line 392 and Lines 396-398).

**Another question for the authors. Do community members eat opossum in anyway? I would mention whether this practice is present and could lead to another source of oral transmission with undercooked meat. In some cultural practices, people will drink raw blood for treatment of aliments. This practice is seen in Mexico with armadillo and other regions like Colombia.** [**https://pubmed.ncbi.nlm.nih.gov/38251211/**](https://pubmed.ncbi.nlm.nih.gov/38251211/)

We thank the reviewer for this comment. Unlike other regions of Latin America and Mexico, opossum meat and by-products, such as blood, are not commonly consumed in Yucatan, thus significantly reducing the risk of transmission of *T. cruzi* through the consumption of meat or blood from infected opossums.

**Summary and General Comments**

**Overall, the manuscript is well written and investigates the importance of the Virginia Opossum in the life cycle of T. cruzi in Merida, Mexico. The work is sound and thorough for research questions poised. I have some suggestions for the manuscript which are listed in the Methods, Results and Conclustions, reports.**

We thank reviewer # 1 for his/her comments.

**One Major revision requests:**

**I would suggest that the authors avoid using “southeastern” as a descriptor of the study region. Southeastern Mexico is a large portion of Mexico, includes the states of Oaxaca, Chiapas, Tabasco, Campeche, Quintana Roo, and the Yucatan. I recommend that the authors state in the title, “... from urban areas of Merida, Mexico...” I would also suggest that the author really focus on the fact this work was conducted in Merida and not associate all urbans areas of southeastern Mexico. This is a very important distinction as other urbans areas may have more or less *T. cruzi* infected *D. virginiana* in the landscape and until the research has been conducted, we will not know the true prevalence in other urban settings.**

We thank reviewer #1 for this comment. We now added that our study was conducted in the metropolitan area of Merida and changed “in southeastern Mexico” to “in the metropolitan area of Merida, Yucatan, in southeastern Mexico”. This more accurately reflects our sampling area. This change has been implemented throughout the manuscript, including the title.

**Minor revision requests:**

**Line 76 and 77, I think the authors meant to state, ...a complex zoonosis that is potentially fatal."**

We thank reviewer #1 for pointing out this error. We made the corresponding edit (Line 78).

***Reviewer: #2***

***Comments to the Author***

**Conclusions**

**In some instances the importance of alternate transmission route is overstated given only single observation of infected milk.**

We thank the reviewer for this comment. We are now more careful not to overstate the impact of our results, as part of the changes made in response to some of the following comments (see below). Particularly, we now clarify at the end of the discussion that, while our results underscore the potential risk represented by *D. virginiana* in non-vectorial transmission, the possibility of a sustained cycle of transmission in total absence of triatomines is rather unlikely in our study area, in part because of the low prevalence observed in the opossum samples with potential role in non-vectorial transmission (Lines 443-449).

**Summary and General Comments**

**Overall:**

**The authors contribute a well-written, well-analyzed investigation of T. cruzi DNA in D. virginiana opossums in the urban setting of Merida, Mexico to provide contemporary data on infection prevalence in this synanthropic species and observations of parasite DNA in milk and anal glad secretion (1 animal each). The work bring awareness to the urban disease ecology of Chagas, an overlooked area as most studies are of rural/impoverished/ranch settings. Opportunistic routes were used for access to animals, including the nuisance animals removed from people’s homes (these were simply picked up manually with no trap, very impressive!); these are a good study population given their epidemiological relevance as they enter homes where people live. The study design, analysis, and interpretation are straight-forward and this adds to a couple recent papers from different geographic areas in the US to bring attention to this reservoir species. Figure 1 is a nice addition to show real**

**photos of processing mammals from the field. I have a few comments for clarification and look forward to seeing this published paper in the future.**

We thank reviewer #2 for his/her comments.

**Major Comments:**

**I feel the manuscript would be improved if the authors can clarify in the Introduction or Discussion what the speculated epidemiological (human health) or ecological significance is, to discern if opossums have vertical** transmission **of T. cruzi? In a region where there are abundant vectors and vectorial transmission, what is the importance to know if vertical transmission also occurs? Perhaps further presentation of the vector community/infection prevalence, and how human case reports compare to other regions of Mexico, would be useful. Given the presence of vectors in homes, I don’t believe it is being suggested that opossums are maintaining parasite in the absence of vectors.**

We thank reviewer #2 for this comment. We have now enhanced the Discussion section by presenting better human cases in the study area (Lines 356-358), the context of home infestation by triatomines and their prevalence of infection in Merida (Lines 375-379), and clarify that, while our results underscore the potential risk represented by *D. virginiana* in non-vectorial transmission, the possibility of a sustained cycle of transmission in total absence of triatomines is rather unlikely in our study area (Lines 443-449).

**Although the overall sample size is over 100 opossums, the sample size of milk and blood from mothers is much lower. In some cases, it seems too much is speculated from the single observation of *T. cruzi* positive milk from infected mother. In the “Conclusions/Significance” section of Abstract, it is stated that detection of T. cruzi in anal gland secretions (plural)….” But only a single secretion tested positive in this study.**

We have revised the sentence in the “Conclusions/Significance” section of the Abstract, changing “anal gland secretions” to “a sample of anal gland secretions” to accurately reflect our findings. We now take more care not to overstate the impact of our results and clarify that, while our results underscore the potential risk represented by *D. virginiana* in non-vectorial transmission, the possibility of a sustained cycle of transmission in total absence of triatomines is rather unlikely in our study area, in part because of the low prevalence observed in the opossum samples with potential role in non-vectorial transmission (Lines 443-449).

**The statistical testing failed to detect a difference among parasite loads among the lactating vs. non-lactating females vs. males (with very large SD that was equal to or greater than the median value), yet the authors often refer to the ‘lower parasite load in females with offspring’ (e.g., abstract line 53). The Discussion (line 374) includes language about a lower parasite load in a study group. That statement is not supported statistically and so the presentation and discussion of these results should be corrected.**

We thank reviewer #2 for his/her comment. We have edited the manuscript to better reflect the results regarding parasite load differences. Specifically, we changed ”had lower parasite load” to “tended to have lower parasite load” (Lines 44-45), “the lower parasite load” for “the observed tendency towards a lower parasite load” (Lines 52-53 and Lines 72-73), ”exhibited the lowest parasite load” to “tended to have lower parasite load” (Lines 410-411), “have lower parasite load” to “tended to have lower parasite load” (Lines 441).

**Minor Comments:**

**Line 30: Change ‘Opossums may have the ability to harbor…’ to ‘Opossums can harbor…’ as this has been shown for some species.**

We have made the change accordingly (Line 29).

**Lines 76-77: Sentence seems to be missing a could words, perhaps insert ‘that is’ as follows: ‘… a complex zoonosis that is potentially fatal’.**

We thank reviewer #2 for pointing out this error. We made the corresponding edit (Line 78).

**Re: the methods for animal processing Was no chemical immobilization used for the opossums? What was the fate of the animals after sample collection? Euthanasia or release details should be provided.**

Chemical immobilization was not employed for the opossums; instead, they were manually restrained during sample collection. After sampling, they were released unless they presented a condition incompatible with relocation. Details are now provided (Lines 179-183 and 196-199).

**Did all the samples that tested positive using the TCZ1/2 PCR, also test positive in the assay to quantify parasite load; and did the all also test positive in the DTU determination assay? Knowing that each assay has a different sensitivity, some clarification on the congruence among assays in this sample set will be useful.**

We thank reviewer #2 for this interesting observation. All blood samples that tested positive with TcZ primers also tested positive in the DTU determination assay. We now rewrote this part in the Results Section, which now reads: “Additionally, DTU determination was possible for all positive blood samples and showed that they belonged to TcI (S1 Table)” (Lines 311-313).

**If you have not already, can you deposit the study sequences (the opossum sequences showing the different haplotypes) into an open access sequence database?**

The opossum sequences have now been submitted to Genbank (Submission ID 2885440), with the following accession numbers assigned to each sequence: CytbHap1 (PQ516272), CytbHap2 (PQ516273), CytbHap3 (PQ516274), CytbHap4 (PQ516275), CytbHap5 (PQ516276), CytbHap6 (PQ516277), and CytbHap7 (PQ516278). We have added this information in the manuscript (Lines 241-242 and 303–304). Note that the Supplementary S2 file also contains all the haplotypes identified in the study.

**Lines 280-282 and Figure 2: Since quantification was based on comparison to standard curve of DNA, perhaps clarify that this is parasite equivalents of DNA rather than parasites.**

We thank reviewer #2 for this comment. We have now clarified in lines 253-254, 344-345, and in Figure 2 that quantification was based on parasite DNA equivalents, derived from the standard curve, rather than direct parasite counts. These adjustments allowed enhancing clarity and accuracy in the reporting of our results.

**Line 369: change gender to sex, as sex is a biological concept, while gender is a social and cultural concept.**

We have made the change accordingly (Line 436).
